# Conflicted and confused? Health harming industries and research funding in leading UK universities

**DOI:** 10.1136/bmj.n1657

**Published:** 2021-07-27

**Authors:** Jeff Collin, Alex Wright, Sarah Hill, Kat Smith

**Affiliations:** 1 Global Health Policy Unit, School of Social and Political Science, University of Edinburgh, Edinburgh, UK; 2 SPECTRUM Consortium (Shaping Public Health Policies to Reduce Inequalities and Harm), UK; 3 School of Social Work and Social Policy, University of Strathclyde, Glasgow, UK

## Abstract

**Jeff Collin and colleagues** review how the UK’s leading universities deal with research funding from health harming industries and call for more effective governance of conflicts of interest

University researchers face growing expectations to engage with commercial sources of funding. This pressure is likely to increase in the context of the covid-19 squeeze^1^ and, in the UK, both Brexit and a research impact agenda promoting external collaboration.^2^ Alongside this, there are efforts to reduce conflicts of interest in research involving pharmaceutical and medical device companies,^3^ and policies rejecting tobacco industry funding.^4^ Yet limited attention has been paid to funding from other health damaging industries such as alcohol, gambling, and ultra-processed food and drink. How well are universities equipped to manage such conflicts of interest?

## Identifying conflicts

Research partnerships with business are often promoted as a panacea, contributing much needed resources while supporting important research into societal challenges.^2^ The UK government^5^ and its public research funding agency, UK Research and Innovation (UKRI),^2^ are both enthusiasts; UKRI has encouraged industry collaboration to help restore the UK economy in the wake of covid-19^6^ and promoted the co-production of health innovations.^2^ The publicly funded UK Prevention Research Partnership, an initiative to reduce non-communicable diseases, similarly encourages “engage[ment] with industry in the business of prevention.”^7^

Incentivising private sector partnership in population health research assumes that commercial interests are compatible with the goals of researchers and policy makers. Yet these interests often diverge,^8^ raising important questions about how research institutions should manage interactions with businesses, especially those whose activities entail negative health effects. The global burden of non-communicable disease is substantially driven by producers, marketers, and retailers of unhealthy commodities,^8^ raising questions about how engagement with such companies can produce effective and ethical health research.^9^ ^10^

Similar questions apply to research relationships with industries such as gambling, pesticides, defence, and fossil fuels, and for companies linked to human rights abuses, bribery, and corruption. Even manufacturers of potentially beneficial products—notably pharmaceuticals— have commercial objectives that don’t consistently align with health goals, creating potential conflicts of interest in research relationships.^11^ Appropriate collaboration with the commercial sector thus requires research institutions to develop frameworks for identifying and managing conflicts of interest. Yet attention to such issues has been strikingly limited.

## Focus on individuals

In 2014, the UK government commissioned a review of research collaborations between businesses and universities.^12^ The resulting Dowling review acknowledged that universities need policies on conflicts of interest, but its primary concern was for such policies to “help protect individual researchers who receive funding from industry against personal criticisms based on misconceptions about the role of industry in this research.”^12^ Universities UK, the umbrella organisation for the sector, developed a concordat to support research integrity, which highlights disclosure of conflicts as part of the “proper conduct” of individual researchers.^13^ Yet, research exploring how researchers actually manage such conflicts finds huge variation, even down to understanding what constitutes a conflict of interest.^14^

Universities’ tendency to hold individuals responsible for managing conflicts of interest can mask systemic problems arising from institutional partnerships with industry.^11^ A recent analysis of drug companies’ role in the opioid crisis criticises universities’ failure to protect research from industry influence.^11^ This echoes longstanding concerns about conflicts of interest in drug research and calls for universities to improve how these are managed.^15^ ^16^ Perhaps surprisingly, there has been much less discussion of conflicts arising in research funded by industries whose products are consistently harmful to health, although these are at least equally pressing.

## Inadequate policies 

To better understand how UK universities govern conflicts of interest, we reviewed institutional policies for managing conflicts in funding relationships with industries whose products or activities are known to be potentially damaging to health, referring here to producers of alcohol, arms, ultra-processed food and beverages (including breastmilk substitutes), fossil fuels, gambling products, and tobacco (see supplementary data on bmj.com). We focused on the 24 public UK universities comprising the Russell Group,^17^ since self-identified world class research institutions might be expected to have well developed governance processes, and used publicly available sources.

Although we identified some examples of good practice, overall, universities’ governance processes for managing conflicts of interest are strikingly inadequate (see supplementary data). We were unable to estimate the scale of research funding from health harming industries because of the lack of transparency. Edinburgh is unusual in having published annual lists of research funding sources and sums^18^; most universities do not publicly disclose these data, and some have declined freedom of information requests relating to specific industries.

University conflict of interest policies invariably focus on individual rather than institutional relationships. This is exemplified by Oxford’s policy, which explicitly “places the onus on the individual involved to recognise and disclose activities that might give rise to conflicts of interest … and to ensure that such conflicts are seen to be properly managed or avoided.”^19^ Some frame management of conflicts as part of the researcher’s duty of care to their university, rather than the other way around.^20^ ^21^ In the absence of processes to support researchers in evaluating possible conflicts, this seems both naive and unreasonable; individual researchers are effectively expected to act as investigator, judge, and jury in appraising any conflicts in their projects.

Perhaps more importantly, such policies are fundamentally incapable of resolving tensions between the core activities of a specific business and a university’s mission or values. They ignore the extent to which key conflicts in health research operate at institutional level and lack an equivalent to the definition of institutional conflicts developed by the World Health Organization. By contrast, WHO’s framework for engagement with non-state organisations aims to manage risks arising from relationships with entities whose interests “are in conflict with WHO’s public health policies, constitutional mandate and interests.”^22^

## Tobacco industry funding: the (partial) exception

Conflicts of interest are partially acknowledged in other governance documents, notably those concerning tobacco industry funding, where university practices have been shaped by Cancer Research UK (CRUK), one of the UK’s leading funders of health research. CRUK’s commitment to avoiding tobacco industry links in funding relationships is reflected in a joint protocol with Universities UK, stating that universities would not wish to lend “support to an industry whose products caused serious damage to health” (while remaining silent on industries with comparable effects).^23^

The protocol outlines principles governing research funding, including requirements that universities develop criteria for accepting research funding and “normally reveal the source of funds for research.”^4^ Yet even for tobacco, university policies are typically underspecified. Minimalist assertions that a university “does not accept research funding from the tobacco industry”^23^ leave many unanswered questions. Do existing policies preclude funding from initiatives like the Foundation for a Smoke-Free World, which supplies grants of $80m (£58m; €68m) a year based on revenue from cigarette giant Philip Morris?^24^ Do they apply to manufacturers of e-cigarettes and other nicotine devices, or only when these are owned by tobacco companies? Does rejection of funding extend to philanthropic donations or teaching support through scholarships or placements?

Some such questions are covered in the more expansive policies adopted by a handful of UK universities. York indicates that it won’t engage in any kind of relationship with tobacco companies,^25^ while Southampton also specifies criteria for assessing whether companies constitute part of the tobacco industry.^26^ Policies from Southampton and Bristol suggest a broader set of principles on which to base funding decisions.^26^ ^27^ Yet these policies offer little specific guidance to researchers, ethics review panels, or university officials.

## Inadequate institutional guidance for managing relationships 

Few universities offer any guidance on funding relationships with industries other than tobacco. Bristol broadly states that it won’t accept funding from sources whose aims are contrary to its objectives and interests, though it cites only tobacco.^27^ Some do now consider specified sources of funding in their ethics review procedures; York states that proposals involving the defence sector may be assessed by its ethics review panel,^25^ while the London School of Economics escalates ethics review of proposals involving “‘caution’ industries” including arms, tobacco, fossil fuel, pornography, and gambling.^28^ Nottingham offers guidance on ethical fundraising, stating that it will not “ordinarily accept philanthropic donations from organisations where the major part of their business” involves arms manufacture and sale to military regimes, tobacco products, or explicit environmental damage.^29^

Strikingly, no university made any reference to managing interactions with the food industry, despite evidence of systematic bias in industry funded research^30^ and concern that uncertainty around appropriate relationships inhibits effective collaboration.^31^

One aspect of research governance that has seen innovation is the increasing adoption of divestment policies, often responding to student-led campaigns targeting fossil fuels or arms manufacturers.^32^ ^33^ Yet there is no sign of an equivalent wave of pressure or policy adoption regarding research income. Indeed, we found no instances of new restrictions on university investments being accompanied by corresponding restrictions on income sources.

## Better conflict of interest processes 

We recognise that questioning universities’ terms of engagement with commercial organisations can raise concerns about academic freedom, especially given heterogeneous research agendas. Close relationships with commercial organisations are unlikely to be regarded as entailing conflicts of interest within disciplines such as agriculture, engineering, and business, when these interactions are often integral to their work, and health imperialism (imposing a health focused agenda on other sectors) should clearly be avoided. However, it seems appropriate to question the adequacy of existing governance arrangements, building on the precedent of tobacco.

While our primary concern is the potential for unhealthy commodity industries to influence health research and policy, the UK’s Medical Research Council has viewed this deficit in governance as impeding potential for effective engagement with commercial organisations.^31^ Since pressures and incentives for closer financial links between universities and commercial organisations seem unlikely to weaken, there is a clear case for managing such relationships more effectively. Universities also need to consider how their funding relationships affect their crucial societal role, including their research independence and integrity.

While none of the universities reviewed offer a model approach, initiatives in the UK and beyond could help inform more coherent governance (box 1). Such a framework might include the commitment that funding relationships are consistent with a university’s stated mission or values; explicit guidelines to help evaluate and manage the potential risks and benefits of engaging with commercial entities; and, crucially, sufficient transparency on funding relationships to enable their implications to be properly debated and appraised.

Box 1 **Examples of improved governance of conflict of interest in leading universities**

**Assess compatibility with university’s aims and mission: University College London**

“While the guiding principle of UCL’s research funding policy is to generate funds to facilitate research, there are circumstances where it is not appropriate for UCL to accept money from a particular funder, either in general, or for a particular project where such funding might conflict, or be inconsistent, with the aims, objects or activities of UCL, as set out in its mission statement or elsewhere.”^34^

**Apply policies on tobacco industry to other health damaging industries: University of Bristol**

“[T]here are some areas of research where the ethical implications will be particularly important [including] where there is a risk of damage to the environment … where the research is politically or socially sensitive; where there might be a reputational risk to the university.”^27^

**Integrate scrutiny of sources of funding into ethics review process: Durham University**

Durham’s ethics and governance toolkit includes a section on sources of research funding, flagging for full ethical review cases where “a funder or collaborator’s motives are at odds with the university’s ethos and values.”^29^ Areas identified for particular attention include those engaged with or closely connected to arms manufacture, tobacco, alcohol, gambling, or pornography.^35^

**Increase transparency regarding sources of research funding: University of Edinburgh**

Edinburgh has reported on research grants and other sources of income by sector and by source (UK, EU, overseas), including sums from specific companies. In 2015-16, for example, this included £283 750 (€330 000; $390 000) from the US based defence and aerospace company Lockheed Martin, £485 628 from the UK Atomic Weapons Establishment, and £61 030 from the alcohol producer Diageo.^18^

**Develop a decision tree or guidelines to manage engagement with industry: University of Sydney**

The University of Sydney’s Charles Perkins Centre has developed guidelines which clearly start from the desirability of promoting partnerships but which provide clear criteria for assessing the appropriateness of specific collaborations. The approach provides guidance for individual researchers to consider when exploring potential collaborations in a notification of intent process that allows for institutional risk-benefit analysis.^36^

As a first step in advancing more effective governance, universities should be required to routinely disclose their sources of funding.^4^ The current lack of accountability mechanisms creates both an impetus and an opportunity for innovation to stimulate wider change. Medical schools and health faculties should initiate institutional practices that stimulate broader debates. Health charities engaged in research partnerships should question wider funding relationships. And student societies that lobbied for disinvestment from health damaging industries should challenge acceptance of research funding from these same sources.

There is a compelling case for universities to pay more serious attention to institutional processes for managing conflicts of interest. Effective management of engagement with the private sector offers opportunities for tackling global health challenges and promoting sustainability. Failure to do so risks undermining trust in universities as a source of independent inquiry.

**Key messages**

Universities are increasingly relying on commercial sources of funding
Polices for accepting funding from potentially health harming industries are inadequate
Governance focuses on managing conflicts of interest of individuals, neglecting institutional tensions between a university’s mission and the objectives of some commercial funders
Explicit guidelines are needed to identify and manage conflicts of interest with health harming industries, building on experiences in tobacco control
As a first step, universities should be more transparent about funding sources

## Web extra

Extra material supplied by authors

Supplementary data: Search strategies and details of conflict of interest policies

## Notes

Provenance and peer review: Not commissioned; externally peer reviewed.

## References

[1] Davis N. A generation of UK researchers could be lost in funding crisis. *Guardian* 2020 Jun 24. https://www.theguardian.com/science/2020/jun/24/a-generation-of-uk-researchers-could-be-lost-in-funding-crisis

[2] UK Research and Innovation. Corporate plan 2020-21. 2020. https://web.archive.org/web/20210122011042/https://www.ukri.org/wp-content/uploads/2020/10/UKRI-091020-CorporatePlan2020-21.pdf

[3] Moynihan R Bero L Hill S. Pathways to independence: towards producing and using trustworthy evidence. BMJ 2019;367:l6576. 10.1136/bmj.l6576 31796508

[4] Universities UK. CRUK. tobacco industry funding to universities: a joint protocol of Cancer Research and Universities UK. 2004. https://web.archive.org/web/20210119151427/https://www.universitiesuk.ac.uk/policy-and-analysis/reports/Documents/2005/tobacco-industry-funding-to-universities.pdf

[5] UK Government. Industrial strategy white paper. 2017. https://web.archive.org/web/20210202214031/https://assets.publishing.service.gov.uk/government/uploads/system/uploads/attachment_data/file/730048/industrial-strategy-white-paper-web-ready-a4-version.pdf

[6] UK Research & Innovation. £40m grant funding confirmed for business projects tackling post covid-19 global impact. Press release, 20 May 2020. https://web.archive.org/web/20200921003801/https://www.ukri.org/news/40m-grant-funding-confirmed-for-business-projects-tackling-post-covid-19-global-impact/

[7] UKPRP. The UK Prevention Research Partnership (UKPRP): vision, objectives and rationale. 2017. https://web.archive.org/web/20191209003838/https://mrc.ukri.org/documents/pdf/ukprp-background-and-rationale/

[8] Moodie R Stuckler D Monteiro C Lancet NCD Action Group. Profits and pandemics: prevention of harmful effects of tobacco, alcohol, and ultra-processed food and drink industries. Lancet 2013;381:670-9. 10.1016/S0140-6736(12)62089-3 23410611

[9] Collin J Hill SE Kandlik Eltanani M Plotnikova E Ralston R Smith KE. Can public health reconcile profits and pandemics? An analysis of attitudes to commercial sector engagement in health policy and research. PLoS One 2017;12:e0182612. 10.1371/journal.pone.0182612 28886049 PMC5590731

[10] Adams PJ. Moral jeopardy: risks of accepting money from the alcohol, tobacco and gambling industries. Cambridge University Press, 2016 10.1017/CBO9781316118689.

[11] Marks JH. Lessons from corporate influence in the opioid epidemic: toward a norm of separation. J Bioeth Inq 2020;17:173-89. 10.1007/s11673-020-09982-x 32661741 PMC7357445

[12] Dowling A Royal Academy of Engineering. The Dowling review of business-university research collaborations. UK Government, 2015. https://web.archive.org/web/20201217140518/https://assets.publishing.service.gov.uk/government/uploads/system/uploads/attachment_data/file/440927/bis_15_352_The_dowling_review_of_business-university_rearch_collaborations_2.pdf

[13] Universities UK. The concordat to protect research integrity. 2019, https://web.archive.org/web/20210414073959/https://www.universitiesuk.ac.uk/policy-and-analysis/reports/Documents/2019/the-concordat-to-support-research-integrity.pdf

[14] Østengaard L Lundh A Tjørnhøj-Thomsen T. Influence and management of conflicts of interest in randomised clinical trials: qualitative interview study. BMJ 2020;371:m3764. 10.1136/bmj.m3764 33109515 PMC7590918

[15] Booth CM Detsky AS. From the $80 hamburger to managing conflicts of interest with the pharmaceutical industry. BMJ 2019;365:l1939. 10.1136/bmj.l1939. 31053609

[16] Lexchin J Bero LA Djulbegovic B Clark O. Pharmaceutical industry sponsorship and research outcome and quality: systematic review. BMJ 2003;326:1167-70. 10.1136/bmj.326.7400.1167 12775614 PMC156458

[17] Russell Group. Our universities. 2021. https://web.archive.org/web/20210428054944/https://russellgroup.ac.uk/about/our-universities/

[18] University of Edinburgh. Annual review 2015/16: funding. 2019. https://web.archive.org/web/20210505170656/https://www.ed.ac.uk/about/annual-review/1516/funding

[19] University of Oxford. Actions required through the research lifecycle. 2019. https://web.archive.org/web/20210505181101/https://researchsupport.admin.ox.ac.uk/governance/integrity/conflict/actions *(*accessed 05 May 2021)

[20] University of Birmingham. Protocol on conflicts of interest. Human resources, 2010. https://web.archive.org/web/20210505182746/https://intranet.birmingham.ac.uk/hr/documents/public/Professional-Standards/Protocol-on-conflicts-of-interest.pdf

[21] Newcastle University. University policy statement on funding from external sources in ethically difficult cases or from ethically difficult external sources. 2006. https://web.archive.org/web/20210505183230/https://www.ncl.ac.uk/media/wwwnclacuk/research/files/UniversityPolicyStatementonFundingfromExternalSourcesinEthicallyDifficul.pdf

[22] World Health Organization. Framework of engagement with non-state actors (resolution). WHA69.10. 2016. https://web.archive.org/web/20210301032127/https://www.who.int/about/collaborations/non-state-actors/A69_R10-FENSA-en.pdf

[23] University of Edinburgh. University policies. 2019. https://www.ed.ac.uk/research-office/research-integrity/research-integrity-learning/university-policies

[24] van der Eijk Y Bero LA Malone RE. Philip Morris International-funded ‘Foundation for a Smoke-Free World’: analysing its claims of independence. Tob Control 2019;28:712-8. 10.1136/tobaccocontrol-2018-054278. 30242044

[25] University of York. Code of practice and principles for good ethical governance. 2017. https://web.archive.org/web/20210421013346/https://www.york.ac.uk/staff/research/governance/research-policies/ethics-code/

[26] University of Southampton. Statement of responsible collaboration. https://web.archive.org/web/20201201120946/https://www.southampton.ac.uk/~assets/doc/responsible_coll_guidance_tobacco.pdf

[27] University of Bristol. Research governance and integrity policy. 2019. https://web.archive.org/web/20200819153845/http://www.bristol.ac.uk/media-library/sites/red/documents/research-governance/Research%20Governance%20and%20Integrity%20Policy%20V5%20Approved%202019.pdf

[28] LSE. Procedures for the ethical review of grants and donations. 2019. https://web.archive.org/web/20200926201140/https://info.lse.ac.uk/staff/services/Policies-and-procedures/Assets/Documents/proEthScr.pdf

[29] University of Nottingham. Gift acceptance policy and ethical fundraising practice. 2017. https://web.archive.org/web/20200917172630/https://www.nottingham.ac.uk/governance/documents/gift-acceptance-policy-and-ethical-fundraising-practice-july2014-final.pdf

[30] Fabbri A Holland TJ Bero LA. Food industry sponsorship of academic research: investigating commercial bias in the research agenda. Public Health Nutr 2018;21:3422-30. 10.1017/S1368980018002100 30157979 PMC10260999

[31] MRC, NIHR. Review of nutrition and human health research. 2017. https://web.archive.org/web/20210428212707/https://mrc.ukri.org/documents/pdf/review-of-nutrition-and-human-health/

[32] Cardiff University. Revised SRI investment policy (May 2018). https://web.archive.org/web/20210422220307/https://www.cardiff.ac.uk/__data/assets/pdf_file/0006/363723/Investment_Policy_Statement_May_2018.pdf

[33] BBC. QUB to disinvest from fossil fuels after student campaign. *BBC News* 2017 May 16. https://www.bbc.com/news/uk-northern-ireland-39926520

[34] UCL. UCL research funding ethics policy. 2014. https://web.archive.org/web/20210505191510/https://www.ucl.ac.uk/research/integrity/sites/research_integrity/files/research_funding_ethics_policy_may_2014.pdf

[35] Durham University. Research and innovation services—source of funding/ resource or collaborator. 2019. https://web.archive.org/web/20210505191823/https://www.dur.ac.uk/research.innovation/governance/ethics/considerations/funding/

[36] Charles Perkins Centre. Charles Perkins Centre engagement with industry guidelines. University of Sydney, 2016. https://web.archive.org/web/20210505185143/https://www.sydney.edu.au/content/dam/corporate/documents/charles-perkins-centre/CPC%20Engagement%20with%20Industry%20Guidelines.pdf

