# Supplementary material for: Conflicted and confused? Health harming industries and research funding in leading UK universities
Source: BMJ. 2021 Jul 27;374:n1657. doi: 10.1136/bmj.n1657 (PMC8428257; doi:10.1136/bmj.n1657)
Supplement: Supplementary file 1 — Supplementary data: Search strategies and details of conflict of interest policies [file colj060120.ww1.pdf]

Published as supplied by the authors

Supplementary file: Commercial and confused? Health-harming industries and commercial research funding in leading UK universities.

1. Data and research methods underpinning review

Our review aimed to map the existence and range of institutional policies for managing conflict of interest (CoI) in research funding across leading UK universities, focusing in particular on funding relationships with industries whose products or activities are known to have potentially health-damaging impacts. We identified these industries as comprising producers of alcohol, arms, ultra-processed food and beverages (including breast-milk substitutes), fossil fuels, gambling products, and tobacco.

Our review covered the 24 public universities in the UK's Russell Group (Russell Group 2017), on the basis that these are widely regarded and self-identify as world-class research-oriented universities in the UK and might therefore be expected to have the most developed governance processes. We sought publicly-available information relating to their research governance processes by conducting online searches via organisational and external search engines between November-December 2017, with a rapid update in September 2019.

Relevant material was sought via three sources:

1. **Websites of individual universities** (or colleges, in the case of Cambridge and Oxford universities) were searched in order to identify documentation or webpages that might be expected to include information relating to research governance and integrity, funding sources, and investment policies. The text of such documents or webpages was then reviewed in order to identify any statements relating to the identification or management of conflict of interest.
2. **Internet searches** were undertaken using Boolean search strings based on combinations of relevant terms (see examples in Table S1).
3. Results of **Freedom of Information** requests were sought by searching the UK's online Freedom of Information (FoI) repository website (www.whatdotheyknow.com) for each individual university, using the same industry keywords employed in internet searches.

Table S1: Terms used for searching internet and FoI repository (indicative)

Institution	First level search terms (generic)	Second level search terms (specific)
[University Name] AND	Annual report OR<br>Annual review OR<br>Due diligence OR<br>(Private donations OR fundraising policy OR donor policy) OR<br>(Investment OR endowment) OR<br>controversial sourc*	
[University Name] AND	Tobacco industry OR	
[University Name] AND	Alcohol industry OR	*OR AB InBev OR Carlsberg OR Constellation OR Diageo OR E&G Gallo OR Heineken OR NewCo OR Pernod Ricard OR SABMiller*
[University Name] AND	Arms OR armaments OR defence	*OR Airbus OR BAE OR Boeing Defence OR Bombardier OR QinetiQ OR Rolls-Royce*
[University Name] AND	Fossil fuels OR oil	*OR BHP Billiton OR BP OR Exxon Mobil OR Shell*
[University Name] AND	Gambling	
[University Name] AND	Processed food OR sugar industry	*OR Coca Cola OR Kelloggs OR Kraft OR Nestle OR Pepsico*

Inclusion / exclusion criteria. We sought documentation or text published by the universities themselves; such sources comprise the primary data source for our review, and the basis for our subsequent analysis. We did not include information from secondary sources (such as news articles) as part of the review, although these sources were sometimes used to identify primary source material and (in some cases) to contextualise this material.

2. Industry-funded research: Governing conflict of interest

Most Russell Group universities had either a policy or statement regarding CoI in their research governance documentation. The specificity of these statements varied, however. Very few explicitly discussed the issue of acceptance of research funding, and none did so with reference to specific industries. Beyond dedicated CoI policies, issues regarding terms of engagement with commercial sector actors were sporadically addressed across diverse policy and process documents, including ethics review procedures, codes of practice for research integrity, policies on gifts and donations, and statements referring to specific contentious sectors or organisations (principally focused on the tobacco industry).

Thus our review demonstrated both highly variable practice and an overall dearth of governance mechanisms to manage universities' funding relationships with industries whose products or activities are known to have potentially health-damaging impacts (see Table S2).

Table S2. UK Russell Group universities: Presence of publicly-available policies addressing conflict of interest and governing income from health-damaging industries

University	Conflict of Interest policies?			Policies governing income from health-harming industries?	
	CoI Policy Document	*Statements addressing individual CoI*	*Statements addressing institutional CoI*	*Tobacco industry*	*Other health-harming industries*
Birmingham	Yes	Yes	No	No	No
Bristol	Yes	Yes	No	Yes	*Research governance and integrity policy* includes summary statement on policies and guidance regarding controversial sources of funding (tobacco is the only industry specified)
Cambridge	University: No* (Colleges: variable)	(Some Colleges)	(Reference in Downing College COI policy)	No	No
Cardiff	No	Yes (in *Research Integrity and Governance Code of Practice*)	No	No	No
Durham	Yes	Yes	No	Yes	*Ethics & Governance Toolkit* requires ethics review on companies linked to "cautionary topics" (alcohol; tobacco; pornography; gambling; arms; fossil fuel extraction)
Edinburgh	Yes	Yes	No	Yes	No
Exeter	No	Yes (in *Research Ethics Framework*)	No	No	No
Glasgow	Yes	Yes	No	Yes	*General Policy for Externally-Funded Activities* has section on non-permitted sources of funds; specifies only tobacco
Imperial	Yes	Yes	No	No	No
King's College London	Yes	Yes	No	No	No
Leeds	Yes	Yes	No	No	No
Liverpool	Yes	Yes	No	No	No
London School of Economics	Yes	Yes	No	Yes	*Procedures for the Ethical Review of Grants and Donations* also address 'caution industries' including arms, fossil fuels, gambling, and pornography

Manchester	No*	No	No	No	No
Newcastle	Yes	Yes	No	Yes	*Statement on Funding from…Ethically Difficult External Sources* specifies tobacco but no clear policy for other sectors, citing complexity of multi-national companies.
Nottingham	Yes	Yes	No	Yes	*Gift Acceptance Policy and Ethical Fundraising Practice* addresses tobacco, arms, and industries causing ‘explicit environmental damage’
Oxford	Yes	Yes	No	Yes	No
Queen Mary	No	Yes (in *Standards of Business Conduct*)	No	No	No
Queen’s University Belfast	Yes	Yes	No	Yes	No
Sheffield	Yes	Yes	No	Yes	No
Southampton	Yes	Yes	No	Yes	*Statement of Responsible Collaboration* also refers to (unspecified) “other potentially contentious sectors and organisations”
University College London	Yes	Yes	(Implicit in *Research Funding Ethics*)	Yes	No
Warwick	Yes	Yes	(Implicit in *Research Code of Practice*)	No	No
York	No	Yes (in *Code of Practice on Research Integrity*)	No	Yes	*Code of practice and principles for good ethical governance* specifies ethical review for work with the defence sector

*For Cambridge and Manchester, partial exceptions relate to policies addressing conflict of interest in research funded by the US National Institutes of Health.
